# Supplementary material for: Molecular determinants regulating selective binding of autophagy adapters and receptors to ATG8 proteins
Source: Nat Commun. 2019 May 3;10:2055. doi: 10.1038/s41467-019-10059-6 (PMC6499816; doi:10.1038/s41467-019-10059-6)
Supplement: Supplementary file 1 — Supplementary Information [file 41467_2019_10059_MOESM1_ESM.pdf]

Supplementary Information

Molecular determinants regulating selective binding of autophagy adaptors and receptors to ATG8 proteins

M. Wirth et al.

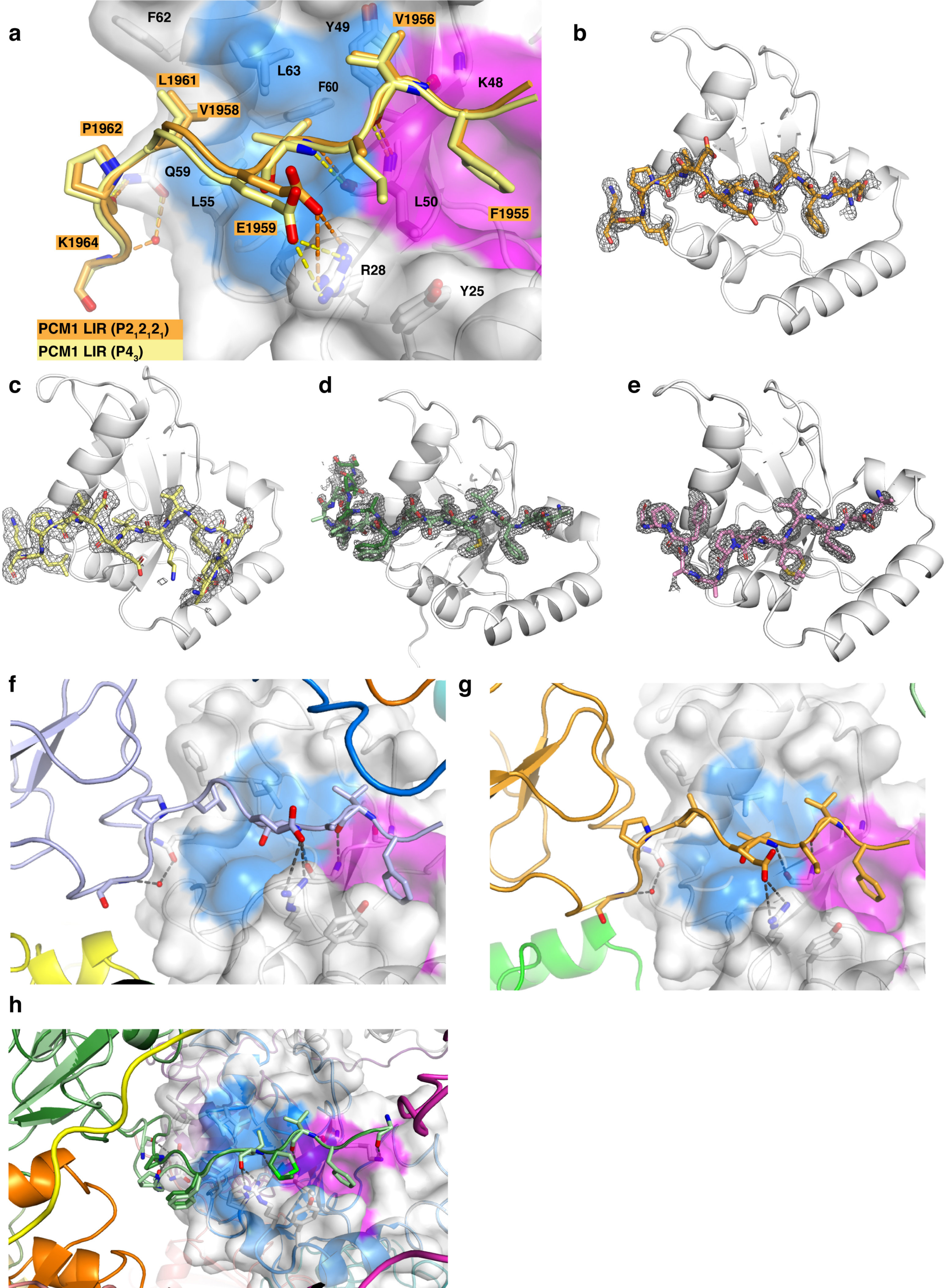

Supplementary Figure 1

Supplementary Fig. 1: PCM1 binds to GABARAP via a C-terminally extended LIR motif. (a) Superposition of the two PCM1$^{1951\text{-}1964}$ LIR-GABARAP structures solved in space group $P2_12_12_1$ and $P4_3$. The PCM1 LIR sequence is shown in orange ($P2_12_12_1$) and yellow ($P4_3$) cartoon with interacting residues depicted as sticks. GABARAP is displayed in white cartoon and transparent surface with hydrophobic pocket 1 and 2 colored in pink and blue surfaces, respectively. (b) to (e) Electron density map of PCM1 LIR $P2_12_12_1$ (b), PCM1 LIR $P4_3$ (c), ATG13 LIR (d), and ULK1 LIR (e) bound to GABARAP. The Fo-Fc omit map of the LIR motifs is contoured at 3.0 σ. GABARAP is displayed in white cartoon and the LIR motif in cartoon and sticks. (f) Crystal packing around the PCM1 LIR $P2_12_12_1$ structure. All the symmetry related molecules within 12 Å are displayed in different colors. (g) Crystal packing around the PCM1 LIR $P4_3$ structure. All the symmetry related molecules within 12 Å are displayed in different colors. (h) Crystal packing around the ATG13 LIR structure. All the symmetry related molecules within 12 Å are displayed in different colors.

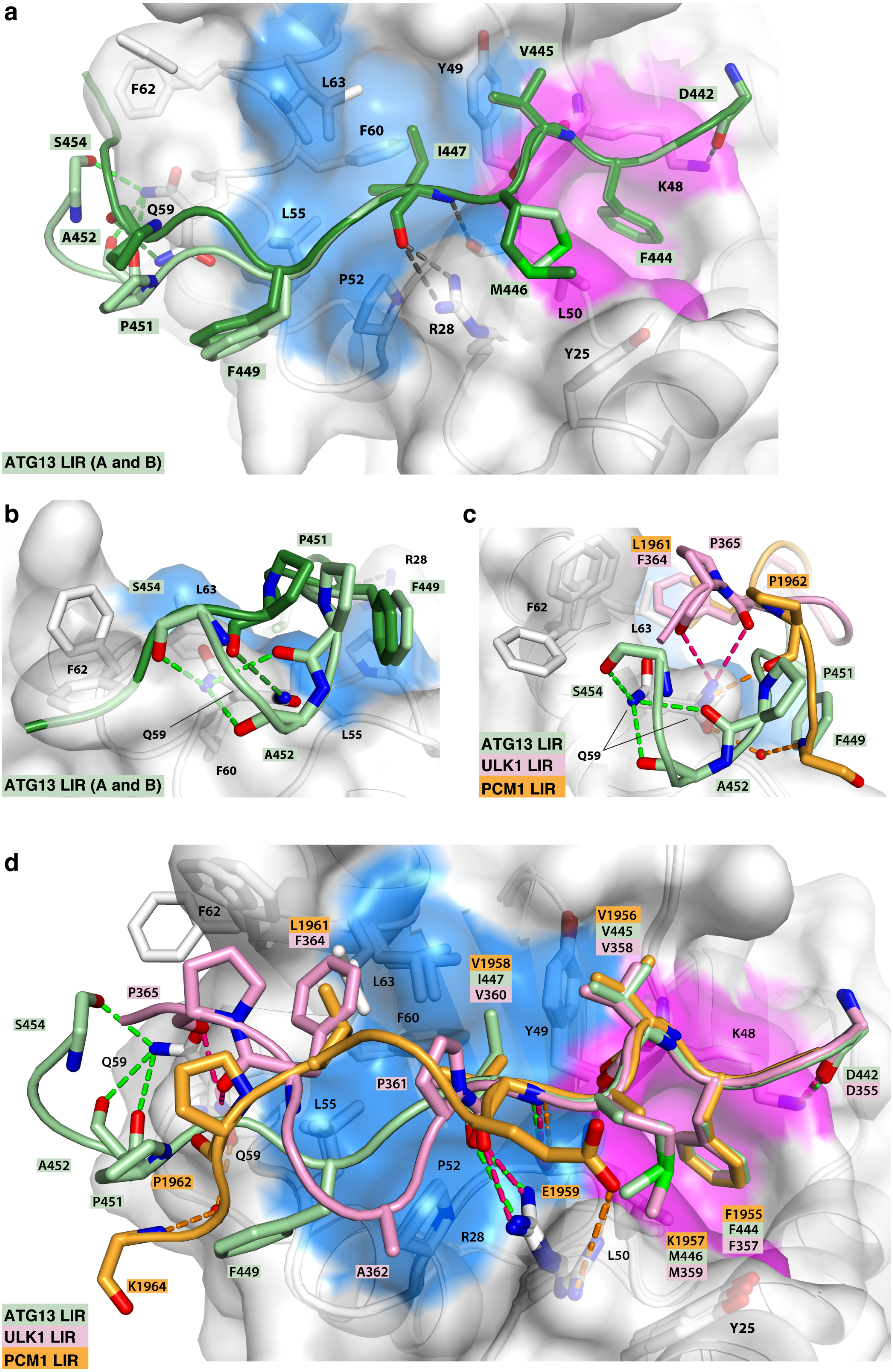

Supplementary Figure 2

Supplementary Fig. 2: ATG13 and ULK1 bind GABARAP via extended LIR motifs. (a) Superposition of the two alternate conformations of $ATG13^{441-454}$ LIR bound to GABARAP. The ATG13 LIR sequences are shown in light green (alternate conformation A) and dark green (alternate conformation B) cartoon with interacting residues depicted as sticks. GABARAP is displayed in white cartoon and transparent surface with hydrophobic pocket 1 (pink) and 2 (blue). (b) Close up view of superposed conformations displaying interactions formed by ATG13 LIR residues in positon X_{5-10}. (c) Close up view of superposed structures of GABARAP bound to ATG13 (light green), ULK1 (pink) and PCM1 (orange) showing interactions of $Q59^{GAB}$, $F62^{GAB}$ and $L63^{GAB}$ with LIR residues in position X_{7-10}. (d) Superposition of structures of GABARAP bound to ATG13 (light green), ULK1 (pink) and PCM1 (orange). In (c) and (d) only the surface of GABARAP from the PCM1:GABARAP structure is displayed in white transparency with HP1 and HP2 colored in pink and blue, respectively.

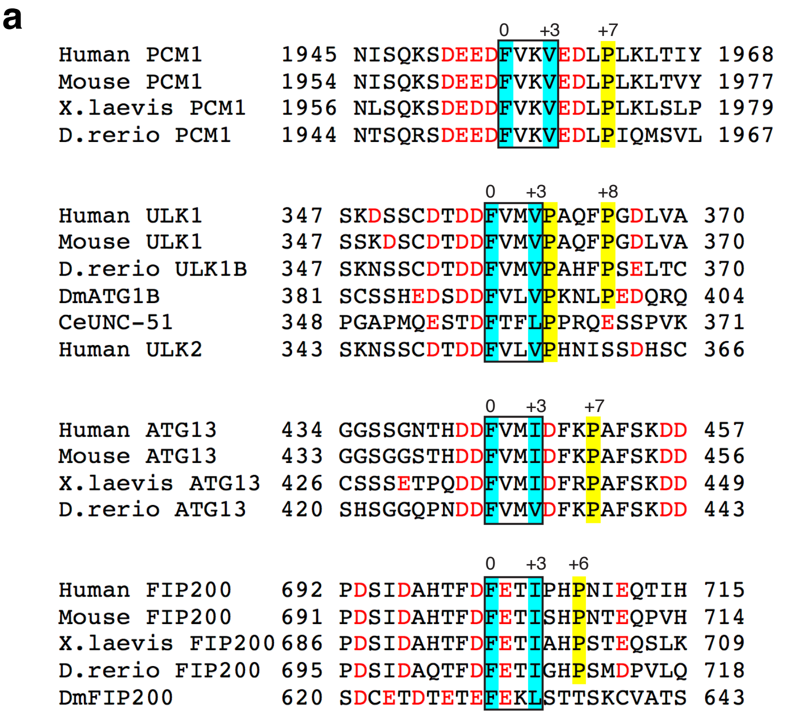

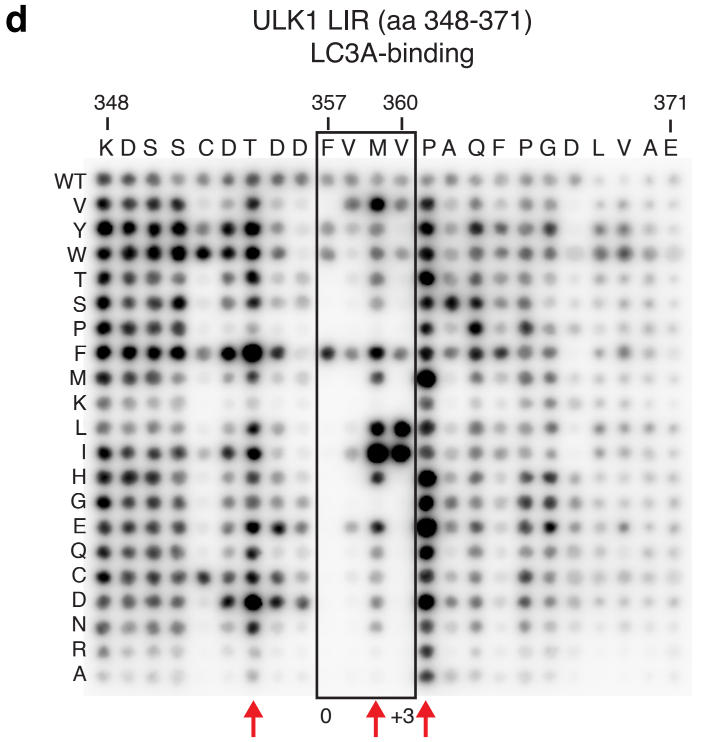

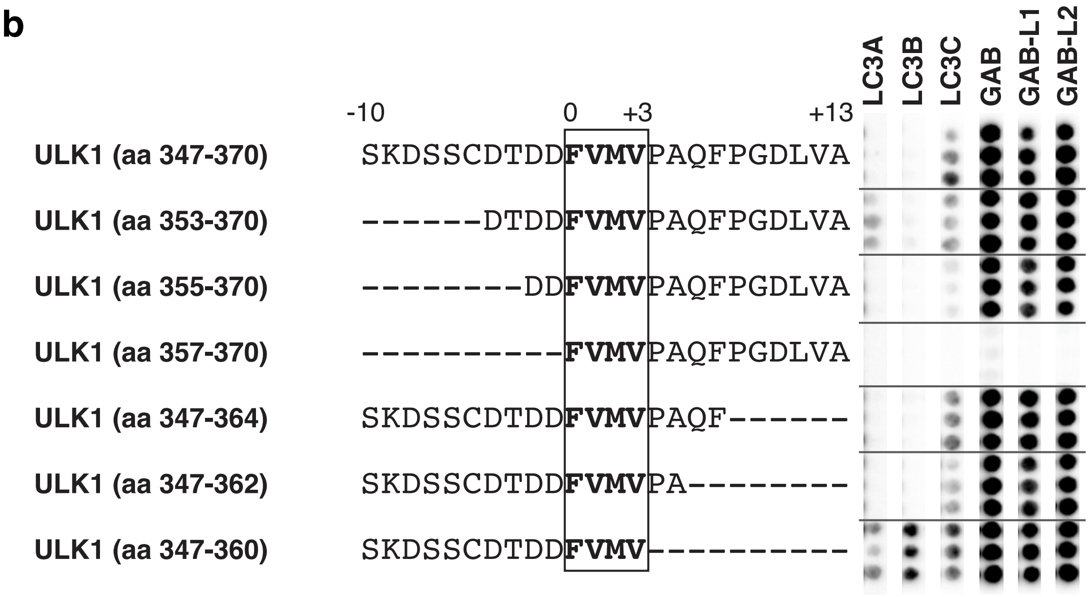

c

	LC3A	LC3B	LC3C	GAB	GAB-L1	GAB-L2	349 357 360 369
ULK1 (aa 349-369)	5.9 ± 0.1	48.2 ± 6.4	2.5 ± 0.3	0.050 ± 0.004	0.048 ± 0.004	0.53 ± 0.05	DSSCDTDDFVMVPAQFPGDLV
ULK1 (aa 353-369)	7.7 ± 1.0	82.0 ± 12.9	1.9 ± 0.2	0.116± 0.009	0.102 ± 0.008	0.58 ± 0.06	----DTDDFVMVPAQFPGDLV
ULK1 (aa 355-369)	407 ± 64.7	494 ± 64.1	7.8 ± 1.4	0.288 ± 0.038	0.243 ± 0.034	2.2 ± 0.3	------DDFVMVPAQFPGDLV
ULK1 (aa 357-369)	3726 ± 907	11247 ± 10315	1156 ± 781	169 ± 42.4	861 ± 153	1499 ± 251	--------FVMVPAQFPGDLV
ULK1 (aa 349-364)	8.4 ± 0.7	68.6 ± 9.4	2.2 ± 0.2	0.166 ± 0.008	0.167 ± 0.009	0.54 ± 0.03	DSSCDTDDFVMVPAQF-----
ULK1 (aa 349-362)	9.2 ± 1.0	76.9 ± 13.1	2.8 ± 0.2	0.174 ± 0.014	0.148± 0.013	0.71 ± 0.05	DSSCDTDDFVMVPA-------
ULK1 (aa 349-360)	2.9 ± 0.3	8.5 ± 0.9	2.2 ± 0.2	0.218 ± 0.020	0.220 ± 0.022	1.1 ± 0.1	DSSCDTDDFVMV---------

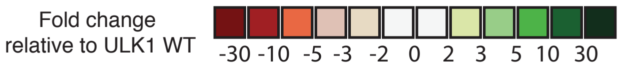

Supplementary Figure 3

Supplementary Fig. 3. The C-terminal region is critical for ULK1 LIR binding specificity. (a) Sequence alignment of PCM1, ULK1, ATG13 and FIP200 LIR motif sequences from various species (X. laevis: Xenopus laevis, D. rerio: Danio rerio, Dm: Drosophila melanogaster, Ce: Caenorhabditis elegans). The core LIR motif is boxed and aromatic and hydrophobic residues in position Θ_0 and Γ_3 depicted in blue. Conserved proline residues are shown in yellow, acidic residues in red. (b) Peptide array of truncated ULK1 LIR peptides incubated with indicated GST-ATG8 protein and immunoblotted with anti-GST. Each peptide is spotted in triplicates. (c) Affinities (Kd values) of truncated ULK1 LIR peptides to ATG8 proteins determined by Bio-Layer Interferometry (BLI). Colour code indicates fold-changes relative to Kd value of ULK1 WT LIR peptide binding to the corresponding ATG8 protein. (data are mean $\pm$ s.d., n=2). (d) Mutational peptide array of 24-mer ULK1 peptide covering LIR motif incubated with GST-LC3A and immunoblotted with anti-GST. Each amino acid position was substituted for every other amino acid.

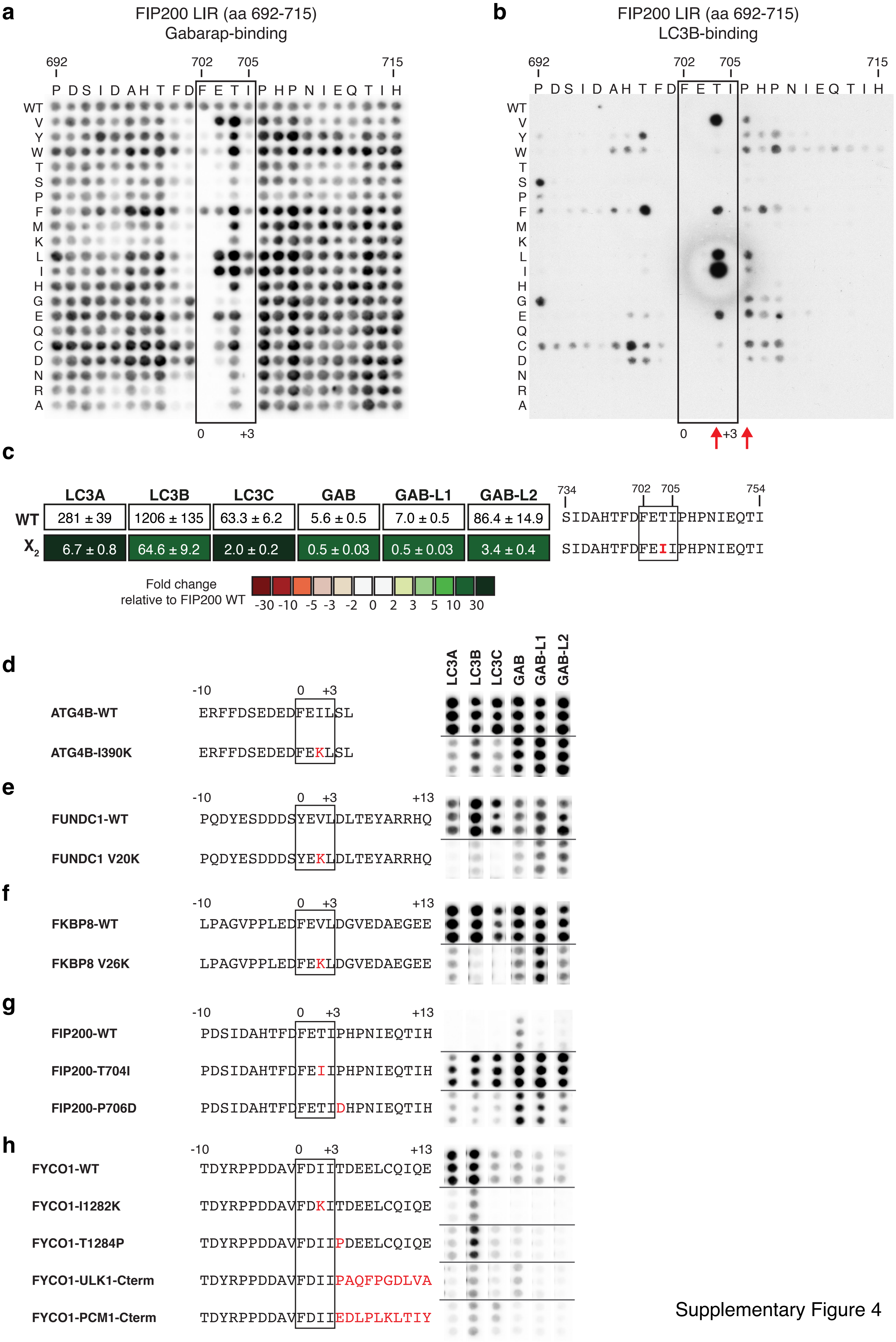

Supplementary Figure 4

Supplementary Fig. 4.: Modulation of X_2 residues changes LIR binding specificity. (a) and (b) Mutational peptide array of 24-mer FIP200 peptide covering LIR motif incubated with GST-GABARAP (a) or GST-LC3B (b) and immunoblotted with anti-GST. Each amino acid position was substituted for every other amino acid. (c) Affinities (Kd values) of FIP200 WT and FIP200 T704I LIR peptides to ATG8 proteins determined by Bio-Layer Interferometry (BLI). Color code indicates fold-changes relative to Kd value of FIP200 WT LIR peptide binding to the corresponding ATG8 protein. (data are mean ± s.d., n=2) (d) to (h) 24-mer peptide array analyses of ATG4B (d), FUNDC1 (e) and FKBP8 (f) FIP200 (g), and FYCO1 (h) LIR peptides containing point mutations incubated with indicated GST-ATG8 protein and immunoblotted with anti-GST. Each peptide is spotted in triplicates. Mutated residues are highlighted in red.

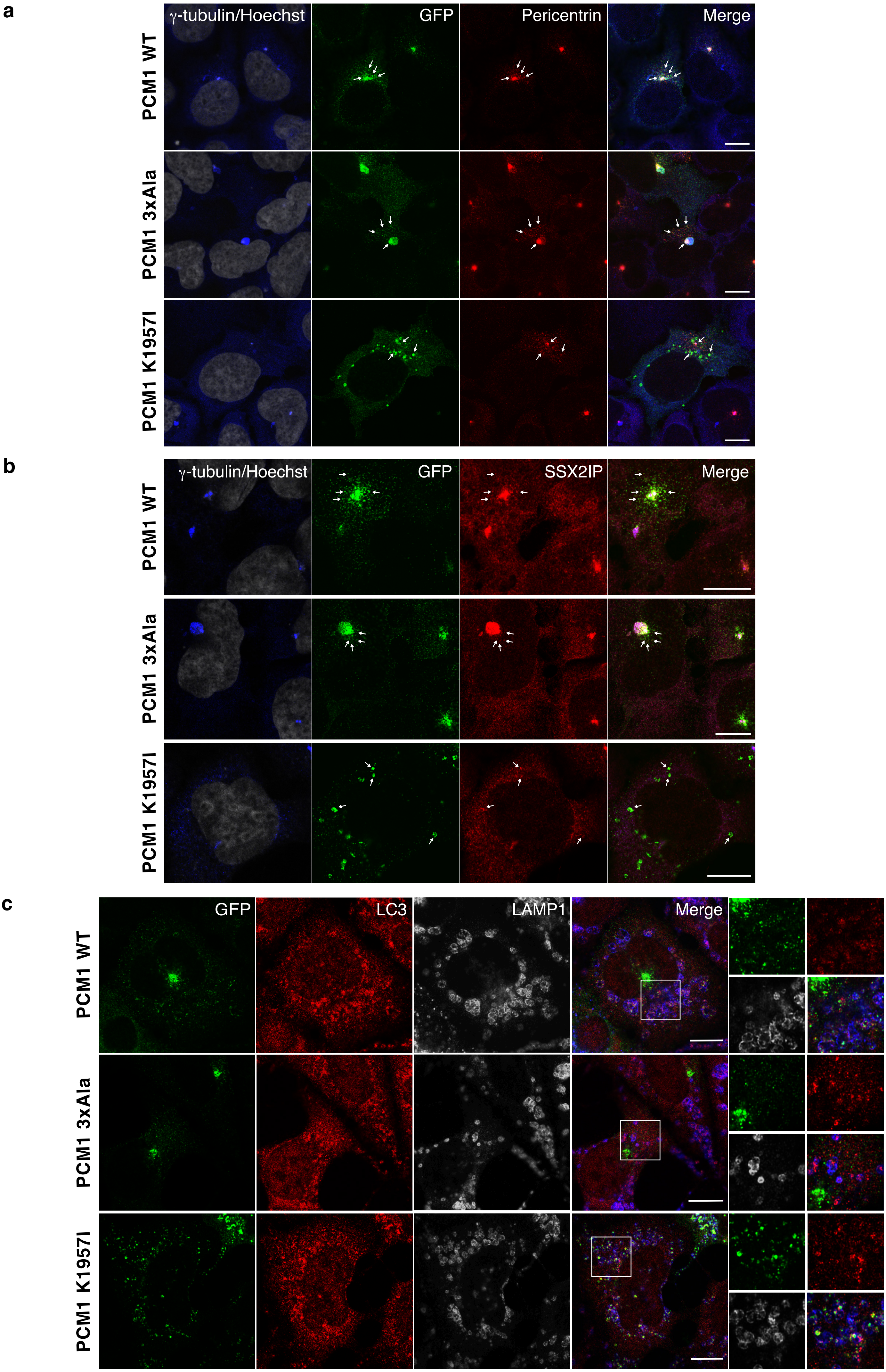

Supplementary Figure 5

Supplementary Fig. 5: PCM1 colocalization with pericentrin, SSX2IP, LC3 and LAMP1. (a) HEK293A cells expressing indicated GFP-PCM1 constructs starved for 2 h in EBSS, fixed and labelled with anti-γ-tubulin, anti-pericentrin and Hoechst. Scale bars represent 10 µm. (b) HEK293A cells expressing indicated GFP-PCM1 constructs starved for 2 h in EBSS, fixed and labelled with anti-γ-tubulin, anti-SSX2IP and Hoechst. Scale bars represent 10 µm. (c) HEK293A cells expressing indicated GFP-PCM1 constructs starved for 2 h in EBSS in the presence of BafA1, fixed and labelled with anti-LC3 and anti-LAMP1. Scale bars represent 10 µm.

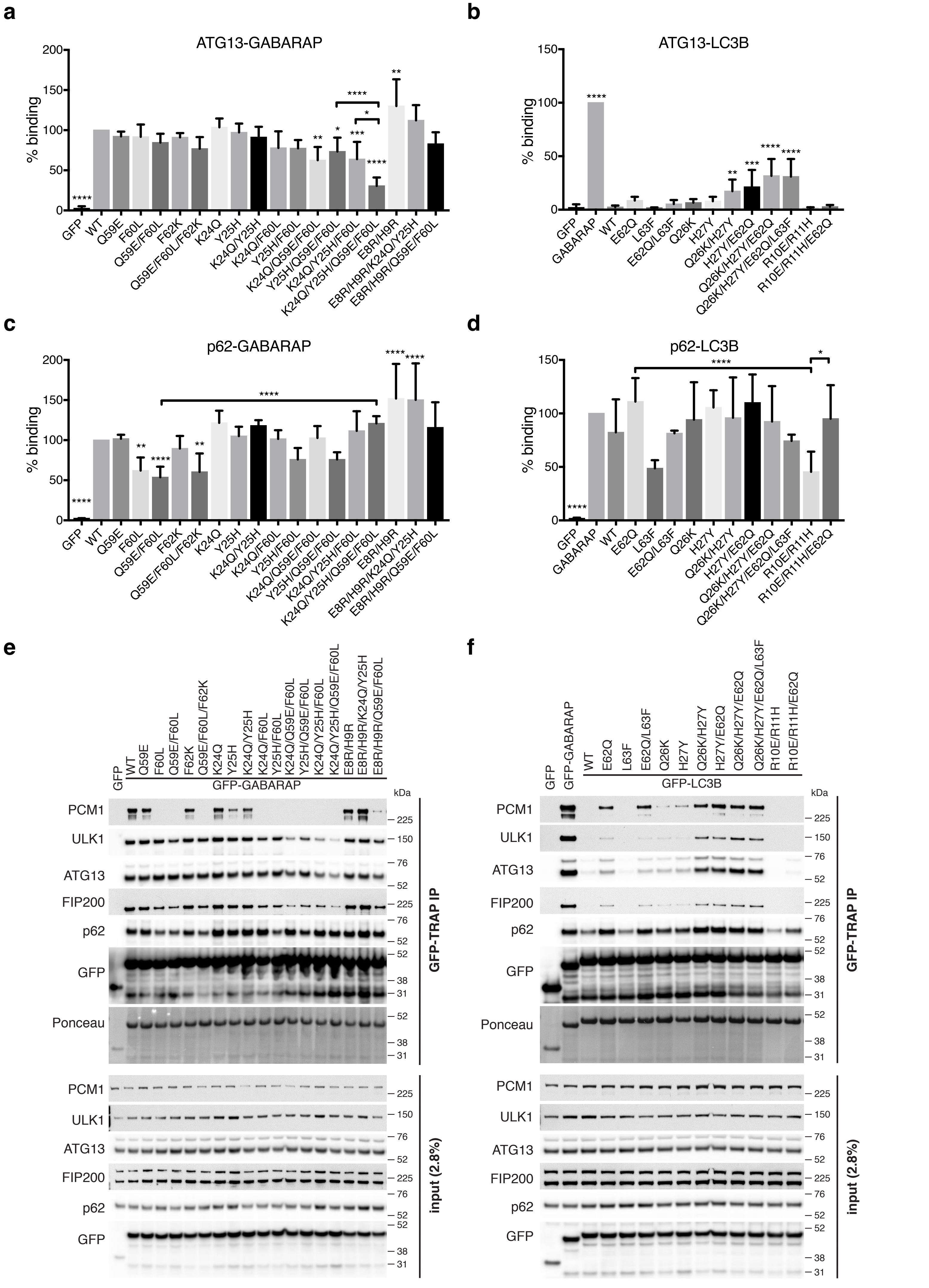

Supplementary Figure 6

Supplementary Fig. 6: Non-conserved GABARAP residues key to selective LIR binding. (a) and (c) Quantification of endogenous ATG13 (a) and p62 (c) binding to indicated GFP-GABARAP constructs expressed and immunoprecipitated by GFP-TRAP from HEK293A cells. (e) Representative immunoblots of GFP-TRAP IP experiments. (b) and (d) Quantification of endogenous ATG13 (b) and p62 (d) binding to indicated GFP-LC3B constructs expressed and immunoprecipitated by GFP-TRAP from HEK293A cells. (f) Representative immunoblots of GFP-TRAP IP experiments. Statistical analysis using One-Way ANOVA test; mean ± s.d.; data from at least three independent experiments. ****$p \leq 0.0001$; ***$p \leq 0.001$; **$p \leq 0.01$; *$p \leq 0.05$.

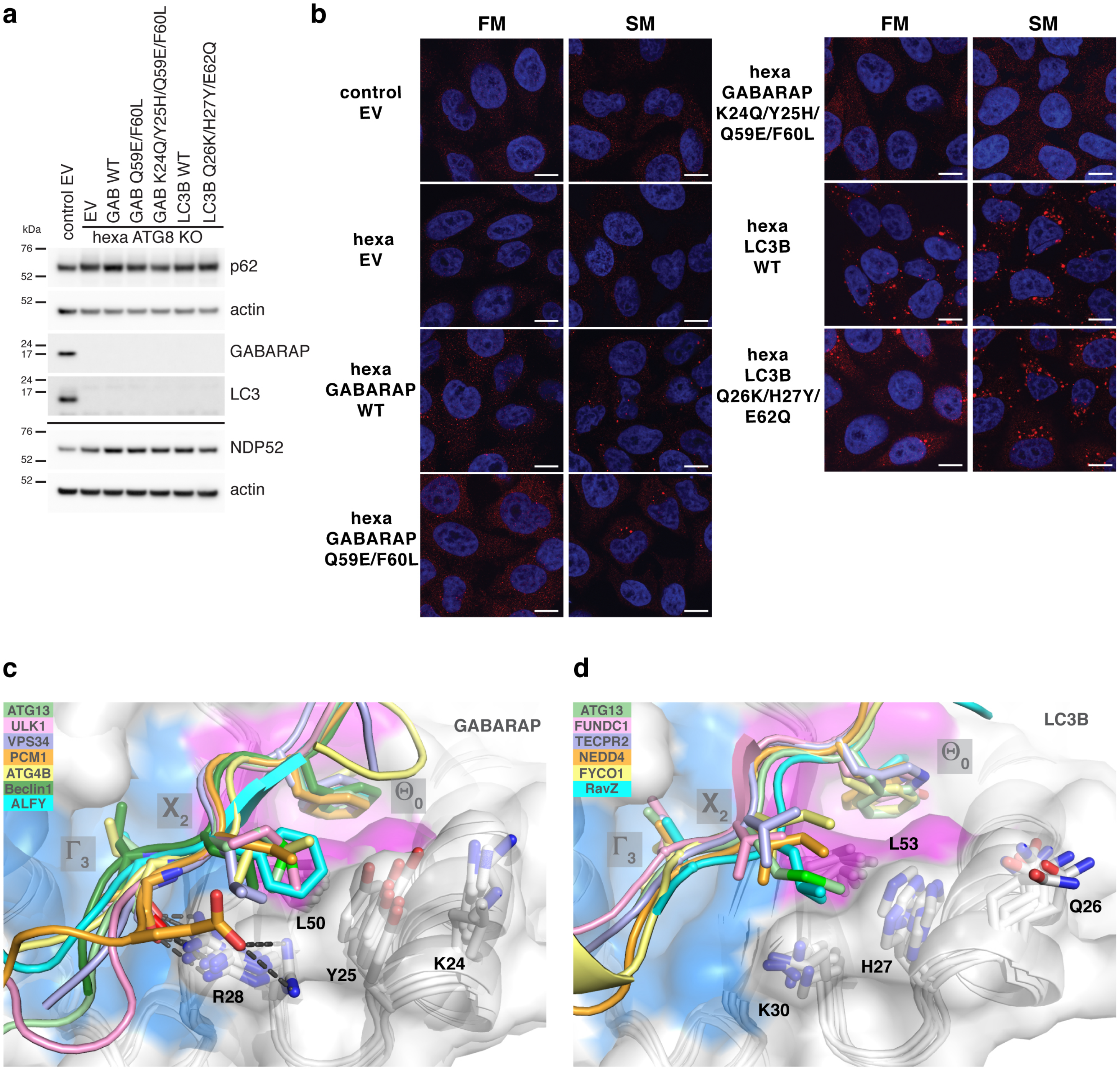

Supplementary Figure 7

Supplementary Fig. 7: Rendering GABARAP more LC3B-like impairs NDP52 degradation. (a) ATG8 CRISPR KO HeLa cell lines stably expressing indicated MYC-ATG8 constructs were grown in full medium without doxycycline prior to lysis and immunoblot. No differences in p62 and NDP52 protein levels between different ATG8 hexa KO HeLa cell lines when MYC-ATG8 construct expression was not induced. (b) ATG8 CRISPR KO HeLa cell lines stably expressing indicated MYC-ATG8 constructs starved for 2h in EBSS, fixed and labelled with anti-MYC and Hoechst. Expression of MYC-ATG8 constructs was induced by 1 µg/ml doxycycline for 6 days. Scale bars represent 10 µm. (c) Structure of the ATG13 LIR bound to GABARAP. The ATG13 LIR sequence is shown in pale green cartoon with residues in position Θ_0, X_2 and Γ_3 depicted as sticks. GABARAP is displayed in white cartoon and transparent surface with hydrophobic pocket 1 and 2 colored in pink and blue surfaces, respectively. GABARAP residues $K24^{GAB}$, $Y25^{GAB}$, $R28^{GAB}$ and $L50^{GAB}$ are shown in white stick. The structures of various LIR motifs bound to GABARAP and containing a hydrophobic residue (except PCM1) in position X_2 were superposed to the ATG13:GABARAP structure for comparison. ULK1 LIR (pink), VPS34 LIR (purple, PDB 6HOG), PCM1 LIR (orange), ATG4B LIR (yellow, PDB 5LXH), Beclin1 LIR (dark green, PDB 6HOJ) and ALFY LIR (cyan, PDB 3WIN). (d) Structure of FYCO1 LIR bound to LC3B (PDB:5WRD). The FYCO1 LIR sequence is shown in pale yellow cartoon with residues in position Θ_0, X_2 and Γ_3 depicted as sticks. LC3B is displayed in white cartoon and transparent surface with hydrophobic pocket 1 and 2 colored in pink and blue surfaces, respectively. LC3B residues $Q26^{LC3B}$, $H27^{LC3B}$, $K30^{LC3B}$ and $L53^{LC3B}$ are shown in white stick. The structures of various LIR motif bound to LC3B and containing a hydrophobic residue in position X_2 were superposed to the FYCO1:LC3B structure for comparison. ATG13 LIR (green, PDB 3WAO), FUNDC1 LIR (pink, PDB 5GMV), TECPR2 LIR (purple, PDB 5DCN), NEDD4 LIR (orange, PDB 5V4K) and RavZ LIR (blue, PDB 5MS6).

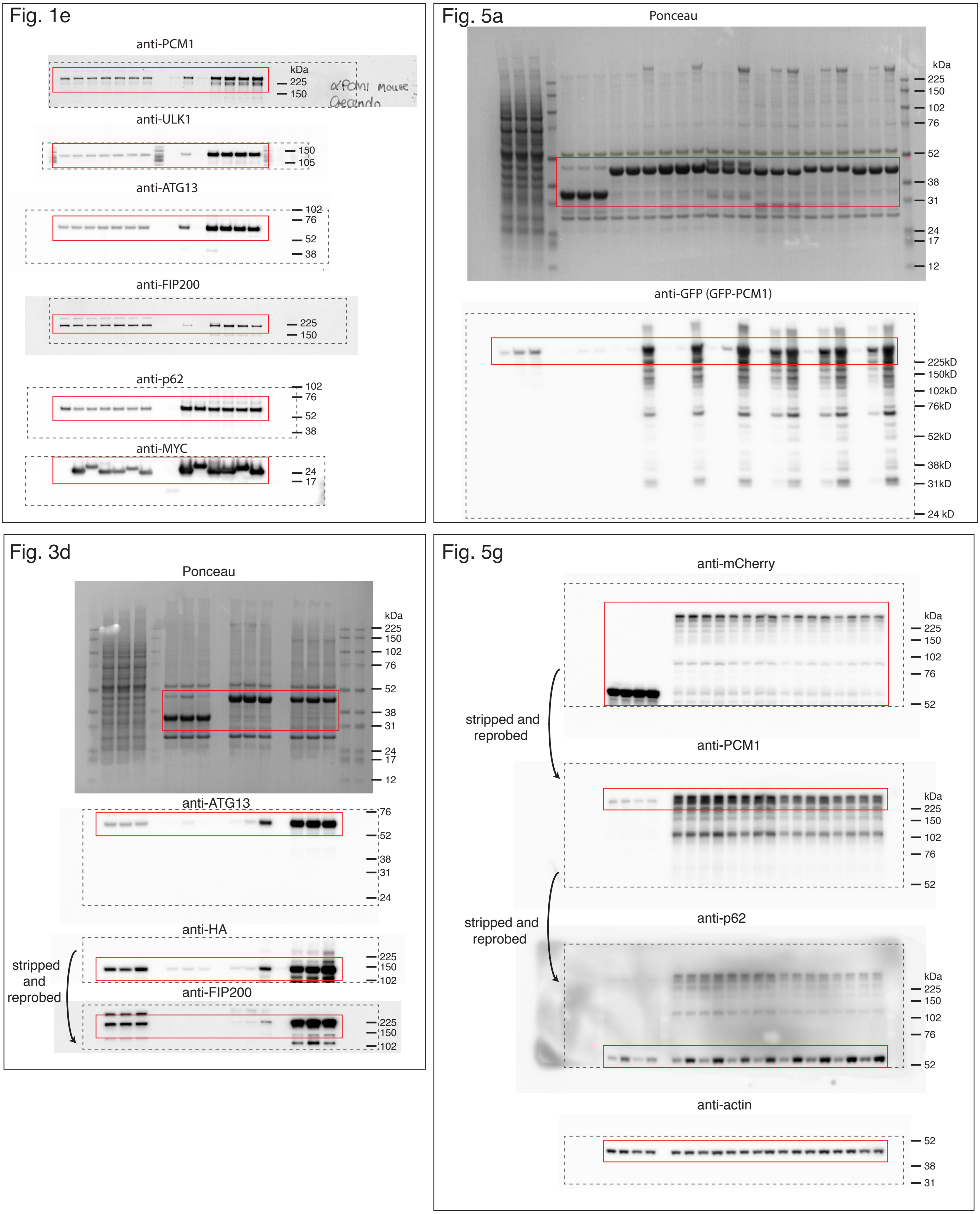

Supplementary Figure 8

Supplementary Fig. 8: Uncropped Western blots.
Full blot is indicated by dashed grey lines, red box shows cropped region.

Fig 7a

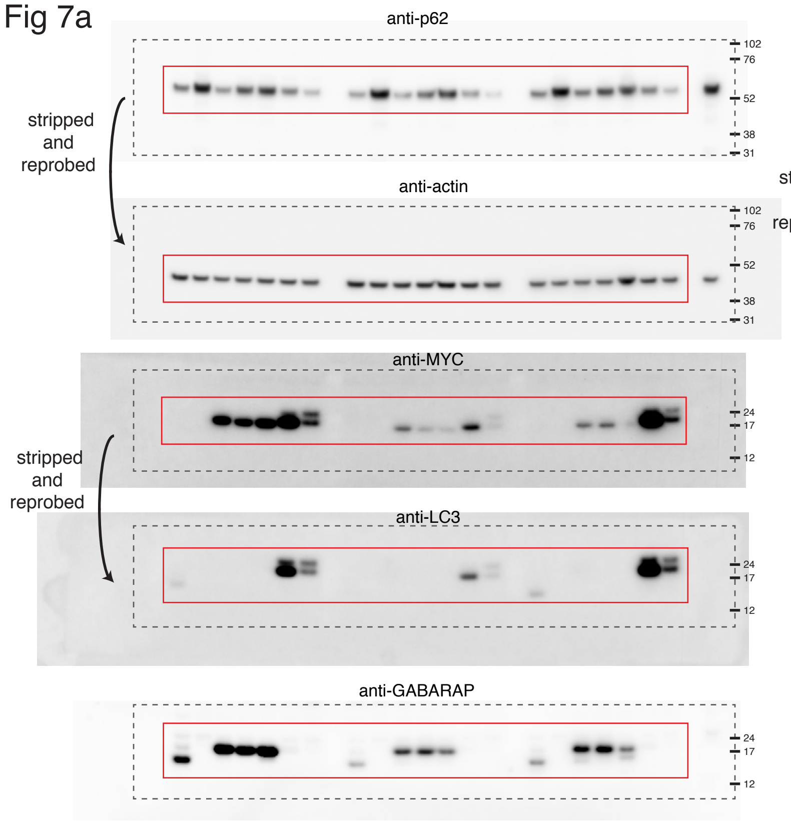

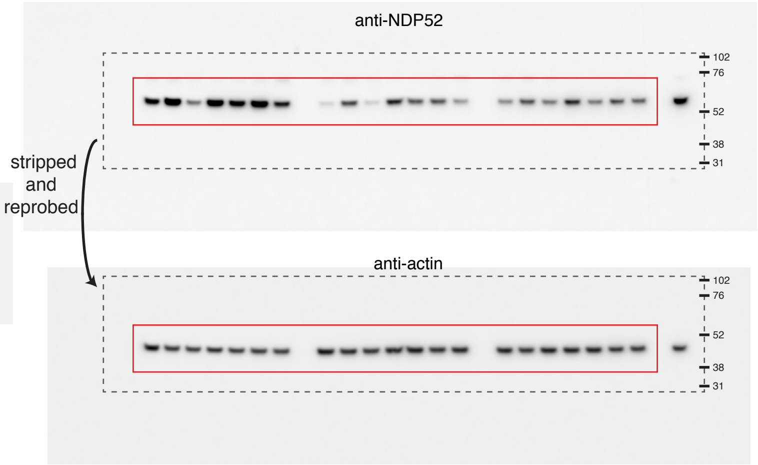

Supplementary Fig. 8: Uncropped Western blots. Full blot is indicated by dashed grey lines, red box shows cropped region.

Supplementary Fig. 6e

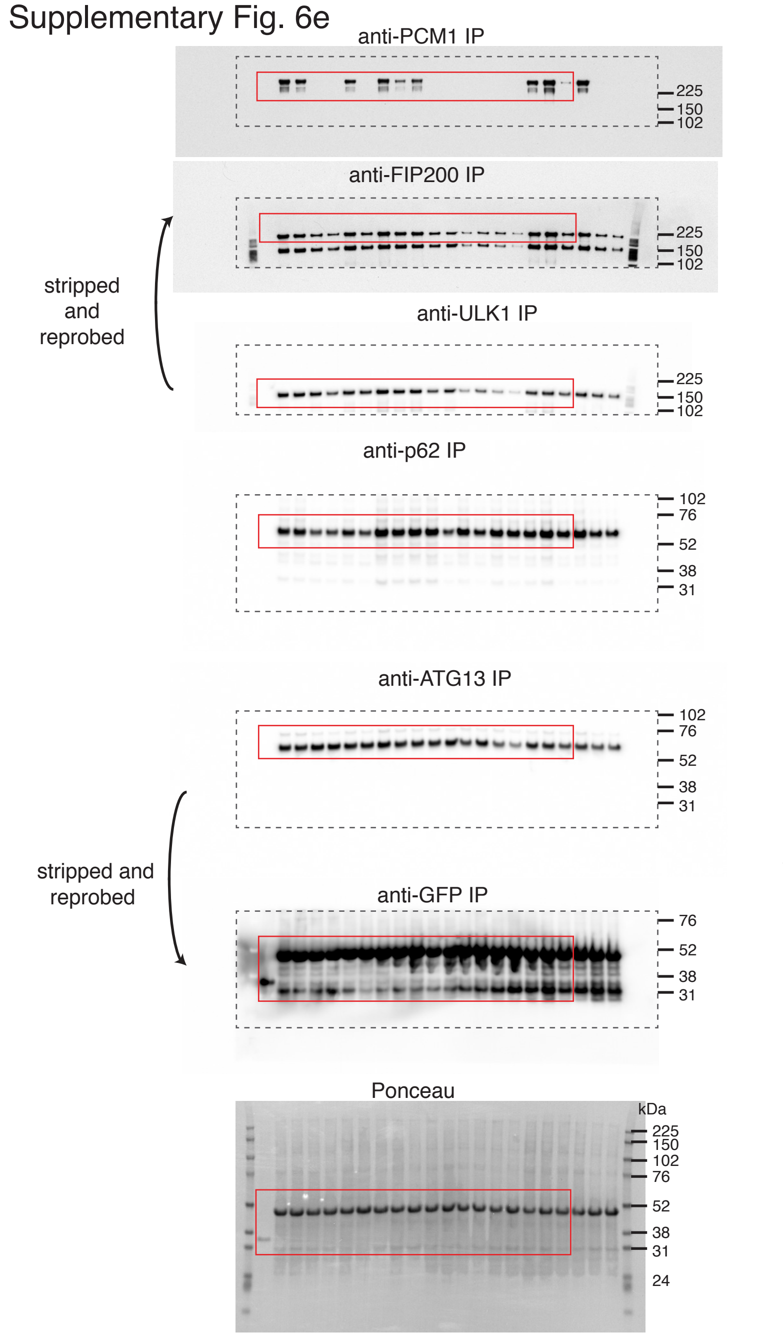

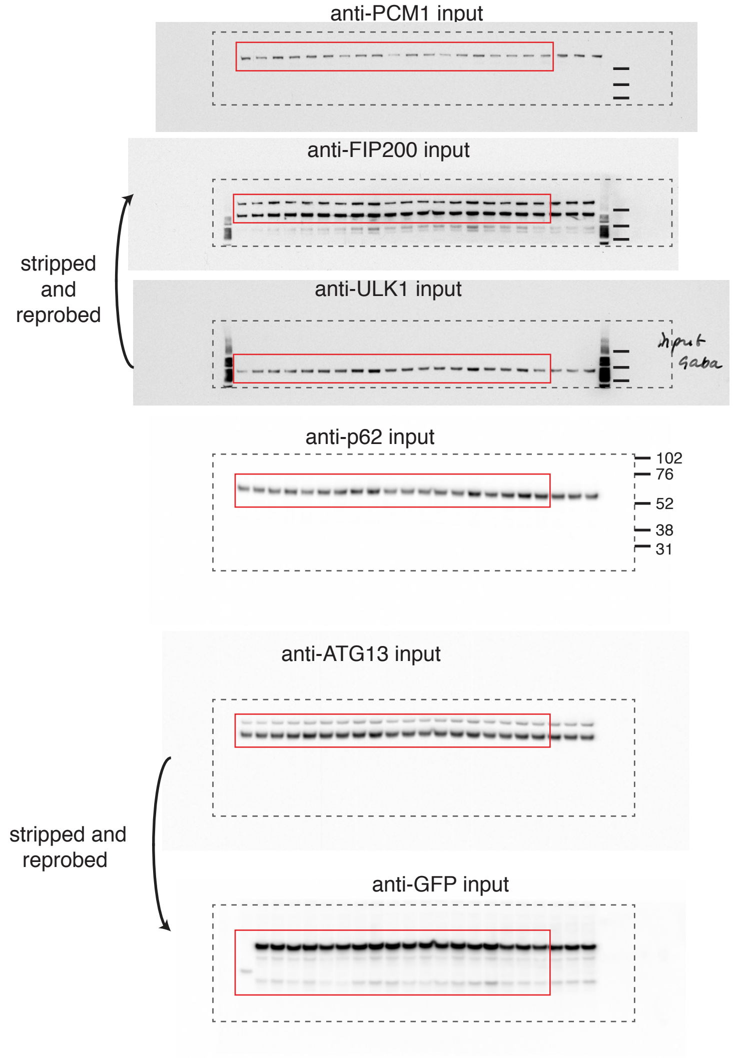

Supplementary Fig. 8: Uncropped Western blots. Full blot is indicated by dashed grey lines, red box shows cropped region.

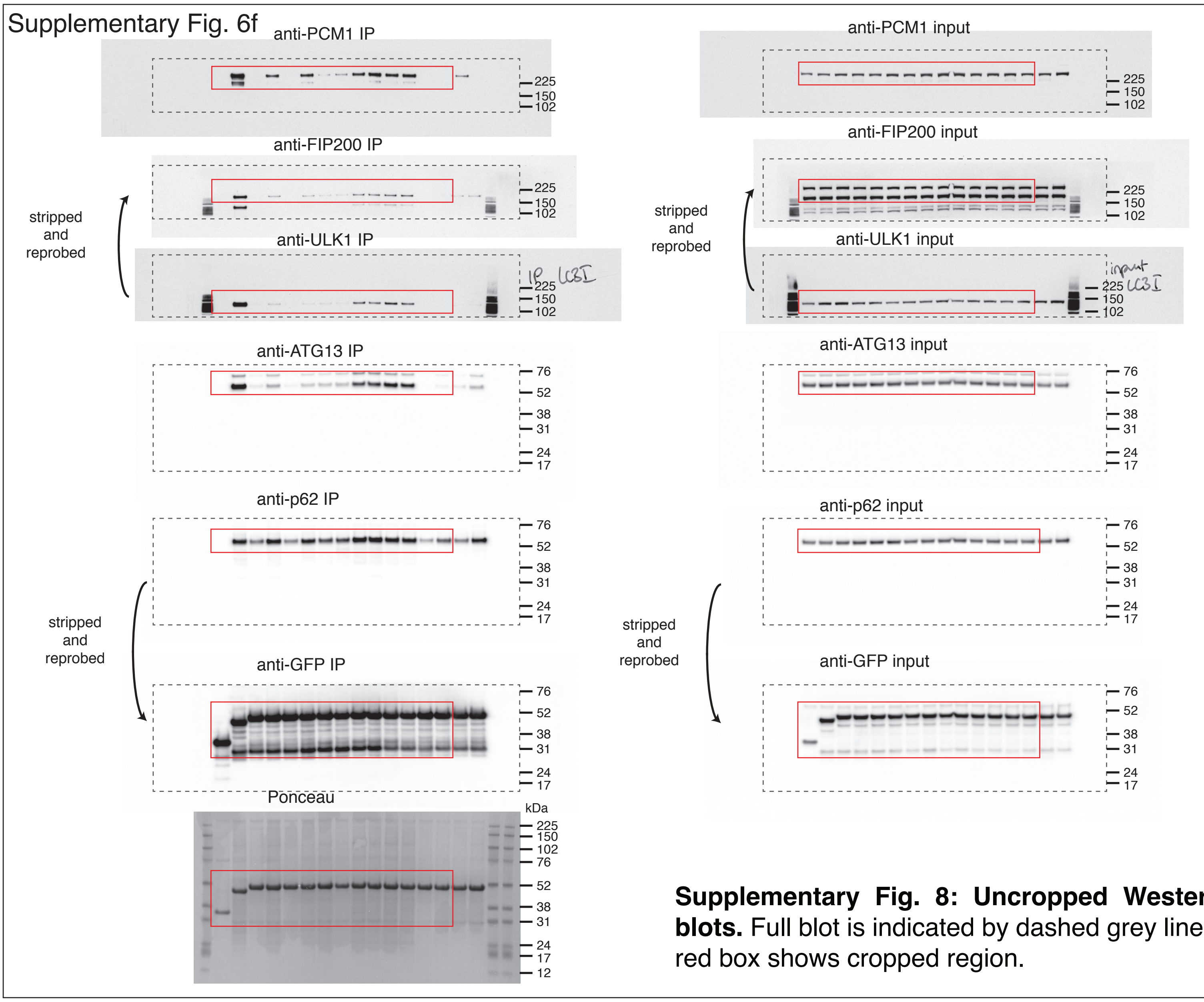

Supplementary Fig. 8: Uncropped Western blots. Full blot is indicated by dashed grey lines, red box shows cropped region.

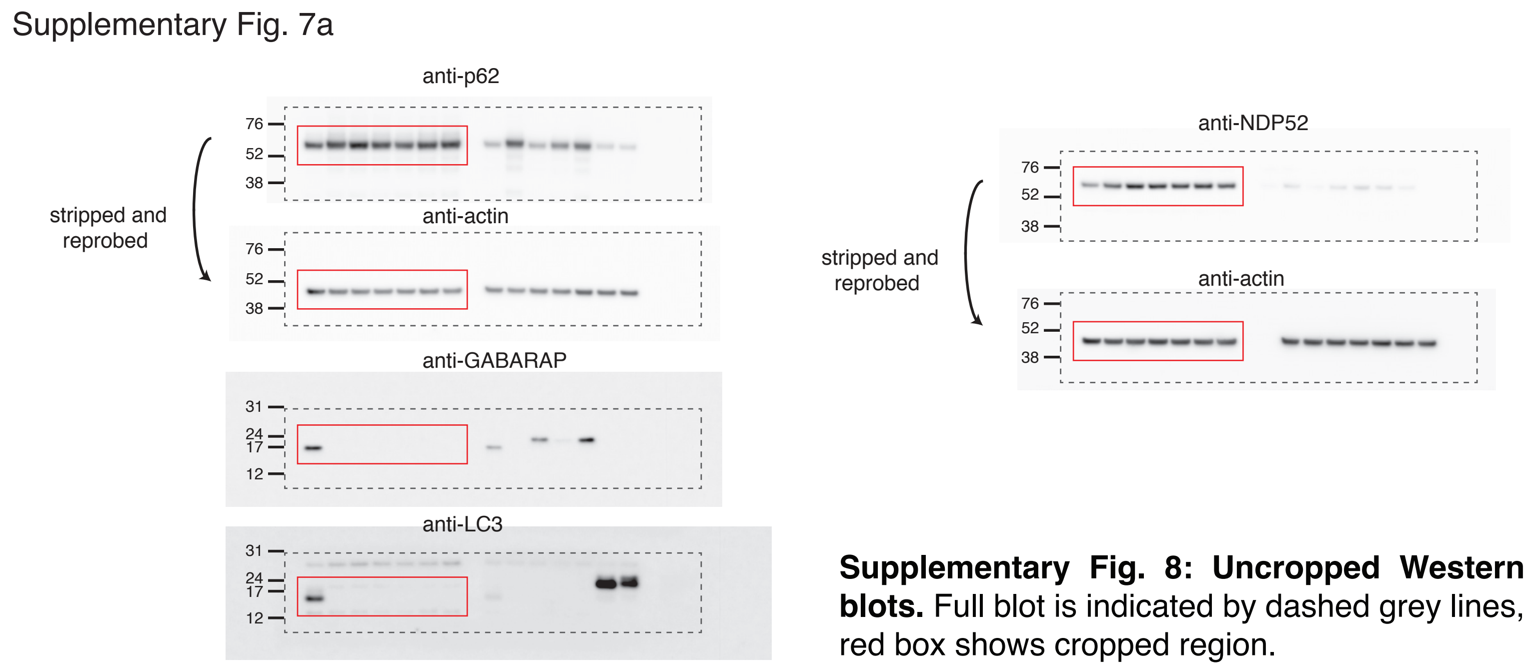

Supplementary Fig. 8: Uncropped Western blots. Full blot is indicated by dashed grey lines, red box shows cropped region.

Supplementary Table 1: Primers used in this study.

Mutagenesis PCR of PCM1 in pEGFP-C2 and pDNOR221 vectors	
**Primer**	**Sequence**
EGFP-PCM1 K1957I (pEGFP-C2) forward	GATTTTGTAATAGTTGAAGATTTACCAC
EGFP-PCM1 K1957I (pEGFP-C2) reverse	TTCTTCATCAGACTTTTGAC
PCM1 FDII (V1956D/K1957I/V1958I) (pDNOR221) forward	GAAGATTTTGACATAATTGAAGATTTACCACTG
PCM1 FDII (V1956D/K1957I/V1958I) (pDNOR221) reverse	TTCATCAGACTTTTGACTTATATTAC
**Gateway cloning of PCM1 WT, 3xAla (D1954A/F1955A/V1958A) or K1957I into pDNOR221 vector**	
**Primer**	**Sequence**
PCM1 forward	GGGGACA AGT TTG TAC AAA AAA GCA GGC TTGatggccacaggaggaggtccc
PCM1 reverse	GGGGACCACTTTGTACAAGAAAGCTGGGTAtcatcatatactctgggctccc acc
**Mutagenesis PCR of pDEST-EGFP-GABARAP and/or pAL-GST-3C-GABARAP**	
**Primer**	**Sequence**
GABARAP Q59E forward	CACAGTTGGTgAGTTCTACTTC
GABARAP Q59E reverse	AGATCAGAAGGCACCAGG
GABARAP F60L forward	AGTTGGTCAGcTCTACTTCTTG
GABARAP F60L reverse	GTGAGATCAGAAGGCACC
GABARAP Q59E/F60L forward	CACAGTTGGTgagcTCTACTTCTTG
GABARAP Q59E/F60L reverse	AGATCAGAAGGCACCAGG
GABARAP F62K forward	TCAGTTCTACaagTTGATCCGGAAGC
GABARAP F62K reverse	CCAACTGTGAGATCAGAAG
GABARAP Q59E/F60L/F62K forward	TGAGCTCTACAAGTTGATCCGGAAGC
GABARAP Q59E/F60L/F62K reverse	CCAACTGTGAGATCAGAAG
GABARAP K24Q forward	GATCCGAAAGCAATACCCGGACCG
GABARAP K24Q reverse	TTCTCGCCCTCAGAGCGG
GABARAP Y25H forward	CCGAAAGAAACACCCGGACCG
GABARAP Y25H reverse	ATCTTCTCGCCCTCAGAG

GABARAP K24Q/Y24H forward	GATCCGAAAGCAACACCCGGACCG
GABARAP K24Q/Y24H reverse	TTCTCGCCCTCAGAGCGG
GABARAP E8R/H9R forward	GAAGTTCGTGTACAAAGAAcgGCgTCCGTTCGAGAAGCGCCGC
GABARAP R28K forward	ATACCCGGACAAGGTGCCGGTG
GABARAP R28K reverse	TTCTTTCGGATCTTCTCGC
GABARAP K24Q/Y25H/R28K forward	CGGACAAGGTGCCGGTGATAGTAGA
GABARAP K24Q/Y25H/R28K reverse	GGTGTTGCTTTCGGATCTTCTCGCC
GABARAP L55V forward	GCCTTCTGATGTCACAGTTGG
GABARAP L55V reverse	ACCAGGTATTTCTTTTTGTC
GABARAP L63I forward	GTTCTACTTCATCATCCGGAAGC
GABARAP L63I reverse	TGACCAACTGTGAGATCAG
**Mutagenesis PCR of pDEST-EGFP-LC3B and/or pAL-GST-3C-LC3B**	
**Primer**	**Sequence**
LC3B E62Q forward	CAACATGAGTcAGCTCATCAAG
LC3B E62Q reverse	ACATGGTCAGGTACAAGG
LC3B L63F forward	CATGAGTGAGTTCATCAAGATAATTAG
LC3B L63F reverse	TTGACATGGTCAGGTACAAG
LC3B E62Q/L63F forward	CATGAGTCAGTTCATCAAGATAATTAG
LC3B E62Q/L63F reverse	TTGACATGGTCAGGTACAAG
LC3B Q26K forward	TATTCGAGAGAAGCATCCAAC
LC3B Q26K reverse	AGTCGGACATCTTCTACTC
LC3B H27Y forward	TCGAGAGCAGTATCCAACCAA
LC3B H27Y reverse	ATAAGTCGGACATCTTCTAC
LC3B Q26K/H27Y forward	TATTCGAGAGAAGTATCCAACCAAAATC
LC3B Q26K/H27Y reverse	AGTCGGACATCTTCTACTC
LC3B K30R forward	CATCCAACCAGAATCCCGGTG
LC3B K30R reverse	CTGCTCTCGAATAAGTCG
LC3B Q26K/H27Y/K30R forward	CAACCAGAATCCCGGTGATAATAGAAC

LC3B Q26K/H27Y/K30R reverse	GATACTTCTCTCGAATAAGTCGGAC
LC3B R10E/R11H forward	CTTCAAGCAGGAGCACACCTTCGAACAAAGAGTAG
LC3B R10E/R11H reverse	GTCTTCTCCGACGGCATG
**Mutagenesis of HA-ULK1 (pCDNA3.1)**	
**Primer**	**Sequence**
ULK1 P361D forward	CGTCATGGTCGACGCGCAGTTTC
ULK1 P361D reverse	AAGTCGTCTGTGTCACAG
ULK1 T354E/M391I/P361D forward	GTCATCGTCGACGCGCAGTTTCCAGGTGA
ULK1 T354E/M391I/P361D reverse	GAAGTCGTCGTCGTCACAGGAAGAGTCCTTGC
**Cloning of MYC-GABARAP WT, Q59E/F60L or K24Q/Y25H/Q59E/F60L and MYC-LC3B WT or Q26K/H27Y/E62Q into pLVX-TetOne-Puro vector**	
**Primer**	**Sequence**
GABARAP AgeI forward	GctgcaggACCGGTGCCGCCACCATGGAACAAAAACTCATCTCAGAAGAGGATCTGTCTggcATGAAGTTCGTGTACAAAGAAGAGC
GABARAP BamHI reverse	CcatgGGATCCTCATCACAGACCGTAGACACTTTCG
LC3B AgeI forward	GctgcaggACCGGTGCCGCCACCATGGAACAAAAACTCATCTCAGAAGAGGATCTGTCTggcATGCCGTCGGAGAAGACCTTC
LC3B BamHI reverse	CcatgGGATCCTCATTACACTGACAATTTCATCCCGAACG
**Cloning of GST-3C-ATG8 proteins into pAL (pGEX-6P2) vector for recombinant protein expression in bacteria**	
**Primer**	**Sequence**
LC3A BamHI forward	CcatgggatccATGCCCTCAGACCGGCCTTTC
LC3A NotI reverse	ATAAGAATGCGGCCGCCTTATCAGAAGCCGAAGGTTTCCTGG
LC3B EcoRI forward	GctgcagggaattcATGCCGTCGGAGAAGACCTTCAAG
LC3B NotI reverse	ATAAGAATGCGGCCGCCTTACACTGACAATTTCATCCCGAACGTC
LC3C BamHI forward	CcatgggatccATGCCGCCTCCACAGAAAATCC
LC3C NotI reverse	ATAAGAATGCGGCCGCCTTACTAGAGAGGATTGCAGGGTCTGTCCT
GABARAP BamHI forward	CcatgggatccATGAAGTTCGTGTACAAAGAAGAGCATC
GABARAP NotI reverse	ATAAGAATGCGGCCGCCTTATCACAGACCGTAGACACTTTCGTCAC
GABARAPL1 BamHI forward	CcatgggatccATGAAGTTCCAGTACAAGGAGGACCAT
GABARAPL1 NotI reverse	ATAAGAATGCGGCCGCCTTATCATTTCCCATAGACACTCTCATCACTG

GABARAPL2 EcoRI forward	GctgcagggaattcATGAAGTGGATGTTCAAGGAGGACCAC
GABARAPL2 NotI reverse	ATAAGAATGCGGCCGCCTTAGAAGCCAAAAGTGTTCTCTCCGCTG
**Cloning of chimera constructs (pAL-GST-3C-LIR motif-GABARAP) for crystallisation**	
**Primer**	**Sequence**
PCM1 LIR NcoI forward	catgggggatgaagaagattttgtaaaagttgaagatttaccactgaaag
PCM1 LIR BamHI reverse	gatcctttcagtggtaaatcttcaacttttacaaaatcttcttcatcccc
ULK1 LIR NcoI forward	CatggggACAGACGACTTCGTCATGGTCCCCGCGCAGTTTCCAGGTg
ULK1 LIR BamHI reverse	gatccACCTGGAAACTGCGCGGGGACCATGACGAAGTCGTCTGTccc
ATG13 LIR NcoI forward	CatggggCATGATGACTTTGTTATGATAGACTTTAAACCAGCTTTTTCTg
ATG13 LIR BamHI reverse	gatccAGAAAAAGCTGGTTTAAAGTCTATCATAACAAAGTCATCATGccc

Supplementary Table 2: Peptides used for BLI affinity measurements.

Peptide	Sequence
ATG13 WT	Biotin-Linker-SSGNTHDDFVMIDFKPAFSKD-Amide
ULK1 WT (aa 349-369)	Biotin-Linker-DSSCDTDDFVMVPAQFPGDLV-Amide
ULK1 (aa 353-369)	Biotin-Linker-DTDDFVMVPAQFPGDLV-Amide
ULK1 (aa 355-369)	Biotin-Linker-DDFVMVPAQFPGDLV-Amide
ULK1 (aa 357-369)	Biotin-Linker-FVMVPAQFPGDLV-Amide
ULK1 (aa 349-364)	Biotin-Linker-DSSCDTDDFVMVPAQF-Amide
ULK1 (aa 349-362)	Biotin-Linker-DSSCDTDDFVMVPA-Amide
ULK1 (aa 349-360)	Biotin-Linker-DSSCDTDDFVMV-Amide
FIP200 WT	Biotin-Linker-SIDAHTFDFETIPHPNIEQTI-Amide
FIP200-T704I	Biotin-Linker-SIDAHTFDFE**I**IPHPNIEQTI-Amide
PCM1 WT	Biotin-Linker-SQKSDEEDFVKVEDLPLKLTI-Amide
PCM1 V1956D	Biotin-Linker-SQKSDEEDF**D**KVEDLPLKLTI-Amide
PCM1 K1957I	Biotin-Linker-SQKSDEEDFV**I**VEDLPLKLTI-Amide
PCM1 V1958I	Biotin-Linker-SQKSDEEDFVK**I**EDLPLKLTI-Amide
PCM1 V1956D/ K1957I/ V1958I	Biotin-Linker-SQKSDEEDF**DII**EDLPLKLTI-Amide
PCM1 E1953A/D1954V	Biotin-Linker-SQKSDE**AV**FVKVEDLPLKLTI-Amide
PCM1 E1959T	Biotin-Linker-SQKSDEEDFVKV**T**DLPLKLTI-Amide
PCM1 P1962A	Biotin-Linker-SQKSDEEDFVKVEDL**A**LKLTI-Amide
FYCO1 WT	Biotin-Linker-YRPPDDAVFDIITDEELCQIQ-Amide
p62-WT	Biotin-Linker-NCSGGDDDWTHLSSKEVDPST-Amide
