# Supplementary material for: Molecular determinants regulating selective binding of autophagy adapters and receptors to ATG8 proteins
Source: Nat Commun. 2019 May 3;10:2055. doi: 10.1038/s41467-019-10059-6 (PMC6499816; doi:10.1038/s41467-019-10059-6)
Supplement: Supplementary file 3 — Reporting Summary [file 41467_2019_10059_MOESM3_ESM.pdf]

nature research

Corresponding author(s): NCOMMS-18-33840-T (Sharon A. Tooze)

Last updated by author(s): Apr 4, 2019

Reporting Summary

Nature Research wishes to improve the reproducibility of the work that we publish. This form provides structure for consistency and transparency in reporting. For further information on Nature Research policies, see Authors & Referees and the Editorial Policy Checklist.

Statistics

For all statistical analyses, confirm that the following items are present in the figure legend, table legend, main text, or Methods section.

n/a	Confirmed	
☐	☒	The exact sample size (*n*) for each experimental group/condition, given as a discrete number and unit of measurement
☐	☒	A statement on whether measurements were taken from distinct samples or whether the same sample was measured repeatedly
☐	☒	The statistical test(s) used AND whether they are one- or two-sided<br>*Only common tests should be described solely by name; describe more complex techniques in the Methods section.*
☐	☒	A description of all covariates tested
☐	☒	A description of any assumptions or corrections, such as tests of normality and adjustment for multiple comparisons
☐	☒	A full description of the statistical parameters including central tendency (e.g. means) or other basic estimates (e.g. regression coefficient) AND variation (e.g. standard deviation) or associated estimates of uncertainty (e.g. confidence intervals)
☐	☒	For null hypothesis testing, the test statistic (e.g. *F*, *t*, *r*) with confidence intervals, effect sizes, degrees of freedom and *P* value noted<br>*Give P values as exact values whenever suitable.*
☒	☐	For Bayesian analysis, information on the choice of priors and Markov chain Monte Carlo settings
☒	☐	For hierarchical and complex designs, identification of the appropriate level for tests and full reporting of outcomes
☐	☒	Estimates of effect sizes (e.g. Cohen's *d*, Pearson's *r*), indicating how they were calculated

Our web collection on statistics for biologists contains articles on many of the points above.

Software and code

Policy information about availability of computer code

Data collection	Azure Biosystems c400 and Amersham Imager 680 user softwares, Octet Red 96 (ForteBio7.1) user software
Data analysis	ImageJ software, GraphPad Prism 7 software, Microsoft Excel (Version 15.39), PyMOL 2.2, PROCHECK, COOT, Phenix, PHASER, Xia2

For manuscripts utilizing custom algorithms or software that are central to the research but not yet described in published literature, software must be made available to editors/reviewers. We strongly encourage code deposition in a community repository (e.g. GitHub). See the Nature Research guidelines for submitting code & software for further information.

Data

Policy information about availability of data

All manuscripts must include a data availability statement. This statement should provide the following information, where applicable:

- Accession codes, unique identifiers, or web links for publicly available datasets
- A list of figures that have associated raw data
- A description of any restrictions on data availability

Atomic coordinates and crystallographic structure factors have been deposited in the Protein Data Bank under accession codes PDB 6HYL (PCM11951-1964-GABARAP, P212121), PDB 6HYM (PCM11951-1964-GABARAP, P43), PDB 6HYN (ATG13441-454-GABARAP), and PDB 6HYO (ULK1354-366-GABARAP). The source data underlying Figures 1e, 3d, 5a, 5g, 7a and Supplementary Figures 6e, 6f, 7a are provided in Supplementary Figure 8. The source data underlying Figures 1g, 4c, 5b-d, 5f, 6, 7b, c and Supplementary Figures 3c, 4c, 6a-d are provided as a Source Data file. All other data that support the findings of this study are available from the corresponding authors upon reasonable request.

Field-specific reporting

Please select the one below that is the best fit for your research. If you are not sure, read the appropriate sections before making your selection.

☒ Life sciences ☐ Behavioural & social sciences ☐ Ecological, evolutionary & environmental sciences

For a reference copy of the document with all sections, see nature.com/documents/nr-reporting-summary-flat.pdf

Life sciences study design

All studies must disclose on these points even when the disclosure is negative.

Sample size	Exact sample size is stated for each experiment in figure legends or methods section.
Data exclusions	NA
Replication	All attempts at replication were successful.
Randomization	NA
Blinding	Blinding was not used in these experiments as observed phenotypes were very strong and it was not helpful for the applied experimental approaches.

Reporting for specific materials, systems and methods

We require information from authors about some types of materials, experimental systems and methods used in many studies. Here, indicate whether each material, system or method listed is relevant to your study. If you are not sure if a list item applies to your research, read the appropriate section before selecting a response.

Materials & experimental systems

n/a	Involved in the study	
☐	☒	Antibodies
☐	☒	Eukaryotic cell lines
☒	☐	Palaeontology
☒	☐	Animals and other organisms
☒	☐	Human research participants
☒	☐	Clinical data

Methods

n/a	Involved in the study	
☒	☐	ChIP-seq
☒	☐	Flow cytometry
☒	☐	MRI-based neuroimaging

Antibodies

Antibodies used	All antibodies used in this study are included and described in the manuscript.
Validation	All commercially available antibodies were validated by manufacturers. Non commercially available antibodies were validated in previous studies which are referenced in the methods section.

Eukaryotic cell lines

Policy information about cell lines

Cell line source(s)	All cell lines were obtained in house from the Francis Crick Institute Cell Services Science Technology Platform or sources indicated in the methods section.
Authentication	None of the cell lines were authenticated.
Mycoplasma contamination	All cell lines were tested for mycoplasma contamination.
Commonly misidentified lines (See ICLAC register)	NA
